# Our Platform

**Published:** 1915-11

**Authors:** 


### Our Platform.

I. SOCIOLOGIC.

Limitation of laws of a criminal nature, to gross crimes, leaving matters of morality and ethics to individual conscience and influence.

Unification and simplification of the machinery of government. The editor personally believes that we have outgrown the need of states and that there should be but one unit, municipal or rural within the national government—this statement not to be understood as interfering with district for educational, fire, police and judicial purposes.

Limitation of local legislation to purely local matters, involving distinctly different principles of feasibility of operation.

Uniform national license, with regard to professional practice, use of highways and all other special forms of control unless strictly local.

Direct and simple taxation, on the basis of income, with such exceptions as are necesary to regulate dangerous industries or for economic reasons aside from the collection of funds.

Against special legislation in the interests of any body, medical or otherwise, even in the interests of the individual unless consented to by a large majority.

A uniform policy with regard to philanthropies, in the sense that any particular philanthropy shall be supported either by taxation or by private benevolences but that there shall be no conflict, and no duplication of expense of administration.

II. MEDICAL.

Against secret fee splitting. For some arrangement which shall secure fair play to both attendant and consultant, specialist and general practitioner, operative, mechanic, scientific and intellectual skill.

Provision for the care of superannuated, sick and needy physicians and members of their families.

Reduction of the medical profession in numbers, mainly by enforcement of educational requirements, but without unreasonable interference with the right to choice of a life work or deliberate destruction of medical schools organized and maintained in good faith.

An efficient ethical code, applying alike to great and small men.

Standardization of ethical relations to allied professions and businesses, in accordance with law and justice.

The maintenance in all professional organizations of the closest practicable approach to the principles of a pure democracy.

Such regulation of educational, philanthropic and other institutions with which the medical profession is necessarily associated, as to conduce to the maximum efficiency of their proper functions and to prevent their exploitation for publicity or other personal ends.

---

Have you ever picked up one of those funny, large page papers that no one seems to buy or sell, but which appear mysteriously in the mail, from time to time, and seen this advertisement: "Would you like to receive lots of mail?" Have you ever laughed at the kind of men who would send in their names just for the sake of getting a lot of pamphlets, sample copies and circulars? If so, shed a tear over the desolate, monotonous, sordid life that needs such diversion and that can be gratified by the envy of the cracker-box brigade about the big stove in the "general store." Then, if you are a manufacturer or dealer, stop and think whether it pays to reach that kind of a man, with your advertising. If he is the one you are after, use the circular letter.

